# Point-of-care capillary HbA1c measurement in the emergency department: a useful tool to detect unrecognized and uncontrolled diabetes

**DOI:** 10.1186/s12245-016-0107-6

**Published:** 2016-02-19

**Authors:** Fernando Gomez-Peralta, Cristina Abreu, Leonor Andreu-Urioste, Ana Cristina Antolí, Carmen Rico-Fontsaré, David Martín-Fernández, Rosa Resina-Rufes, Juan Jesús Pérez-García, Ángela Negrete-Muñoz, Daniel Muñoz-Álvarez, Guillermo E. Umpierrez

**Affiliations:** Endocrinology and Nutrition Unit, Hospital General de Segovia, c/ Miguel Servet S.N., 40003 Segovia, Spain; Department of Internal Medicine, Hospital Nuestra Señora de Sonsoles, C/ Avda. Juan Carlos I, s/n, 05004 Ávila, Spain; Emergency Department, Hospital Nuestra Señora de Sonsoles, C/ Avda. Juan Carlos I, s/n, 05004 Ávila, Spain; General Clinical Research Center, Emory University, 49 Jesse Hill Jr. Dr., Atlanta, GA 30303 USA

**Keywords:** Diabetes, Screening, Point-of-care HbA1c, Emergency medicine, Public health

## Abstract

**Background:**

Inpatient hyperglycaemia and diabetes mellitus (DM) are common and are associated with an increased risk of complications and mortality. The severity of hyperglycaemia determines the rate of complications in patients treated in the emergency department (ED). Our aim was to examine whether determination of the capillary haemoglobin A1c (HbA1c) is a reliable method for detecting unknown diabetes and poor glycaemic control in the ED.

**Methods:**

A prospective observational study was conducted in adult (>18 years) patients treated in a single-centre ED. We compared the results of HbA1c levels measured by Bio-Rad *in2it* point-of-care device on a capillary blood sample and by the hospital laboratory.

**Results:**

A total of 187 ED patients with an average age of 57.1 ± 19.2 years were studied. The mean HbA1c value was 5.78 ± 1.26 % by capillary POC testing and 6.10 ± 1.12 % by the hospital laboratory (correlation = 0.712, *P* < 0.001). A total of 17.1 % of cases had a *prior diagnosis of DM*. The diagnosis of DM (plasma glucose > 126 mg/dL and/or HbA1c > 6.5 %) was made in ten (5.4 %) additional cases (*prior undiagnosed DM*) for a total *prior* DM prevalence of 22.5 % (95 % CI 16.4–28.5 %). Capillary HbA1c detected 11 additional cases of *unknown DM* (5.9 %). A capillary HbA1c value greater than 6 % has a sensitivity of 85.7 % and specificity of 85.3 % for the screening of DM.

**Conclusions:**

Determination of the capillary HbA1c in the ED is a reliable, fast, and simple system for the screening of unknown or uncontrolled DM.

## Background

Hyperglycaemia is a risk marker of morbidity and mortality in the emergency department (ED) and subsequent hospital admission, both in people with and without a history of diabetes mellitus (DM) [1–4]. Detection and management of hyperglycaemia in the ED, however, remains insufficient [5]. Hospital and ED admissions could become windows of opportunity for an early diagnosis of people with diabetes and for improving glycaemic control. Providing information on previous glycaemic control of patients treated in the ED can be useful for stratifying a risk of complications and in tailoring antidiabetic treatment.

Determination of the glycated haemoglobin A1c (HbA1c) value is the best reference measure of glycaemic control in individuals with diabetes. It reflects the glycaemic environment in the past 2 to 3 months. International guidelines recommend measuring the HbA1c value every 3 months in patients that are off target and/or after a therapeutic change, and two annual measurements should be conducted in all patients [6]. In many cases, HbA1c monitoring falls short of the recommended frequency. The HbA1c value is a major indicator to start an intensified medical treatment in patients with DM. In addition, international guidelines have included HbA1c measurement among the methods for diagnosis [6] (Table 1). Silverman et al. proposed HbA1c cut-offs for screening of diabetes in the ED [7]. Using 6 % as the cut-off, the sensitivity of HbA1c was 76.9 % and the specificity was 87.3 %. Even lower HbA1c levels (5.7 %) have been proposed as a useful tool for the screening of prediabetes.

**Table 1:** Criteria for the diagnosis of diabetes in accordance with the American Diabetes Association (ADA) [6]

HbA1c >6.5 %. (The test should be performed in a laboratory using a method that is NGSP certified and standardized to the DCCT assay.)^a^ OR FPG >126 mg/dL (7.0 mmol/L). (Fasting is defined as no caloric intake for at least 8 h.)^a^ OR 2-h PG >200 mg/dL (11.1 mmol/L) during an OGTT. (The test should be performed as described by the WHO, using a glucose load containing the equivalent of 75 g anhydrous glucose dissolved in water.)^a^ OR In a patient with classic symptoms of hyperglycaemia or hyperglycaemic crisis, a random plasma glucose is >200 mg/dL (11.1 mmol/L).	

*DCCT* Diabetes Control and Complications Trial, *FPG* fasting plasma glucose, *NGSP* National Glycohemoglobin Standardization Program, *OGTT* oral glucose tolerance test, *WHO* World Health Organization

^a^In the absence of unequivocal hyperglycaemia, results should be confirmed by repeat testing

In recent years, several methods have become available to quickly and easily measure HbA1c from the capillary blood. There are several available easy-to-use point-of-care systems that can be used in the ED. While they are no substitutes for certified HbA1c measured by the laboratory to confirm the diagnosis of DM, their accuracy is highly satisfactory for estimating the degree of control of DM [8] and detecting undiagnosed diabetes [9] or at-risk subjects [10]. In this study, we propose to test the usefulness of a point-of-care HbA1c system in the ED for the detection of undiagnosed and poorly controlled DM.

## Methods

### Study design

This prospective observational study was conducted at Nuestra Señora de Sonsoles Hospital of Ávila, Spain, a second-level hospital with an ED that covers the needs of a population of nearly 200,000 inhabitants, with approximately 37,000 adult ED visits annually (650 visits a week). We obtained an institutional review board approval prior to initiation of the study.

### Subjects

For seven consecutive days during 2012, informed consent was requested from all adult individuals (over 18 years) who were treated at the ED of Nuestra Señora de Sonsoles Hospital of Ávila. They were asked via a questionnaire whether they had received a prior diagnosis of diabetes. Those that had used corticosteroids in the past 2 months were excluded. The remaining patient’s clinical data was obtained from medical history, including detailed verification of the existence of a prior diagnosis of diabetes or the result of a diagnostic biochemical tests in accordance with the American Diabetes Association (ADA) criteria [6] (Table 1).

A capillary blood sample was taken from all patients in the finger pad. An Accu-Chek Aviva™ (Roche Diagnostic, Indianapolis, IN, USA) glucometer was used for in situ measurement of capillary blood glucose. The capillary HbA1c value was determined in situ by an in2it™ system (Bio-Rad, Hercules, CA, USA). Additionally, a venous blood sample was obtained by venepuncture and was sent to the hospital’s central laboratory for measurement of plasma glucose and HbA1c levels.

### Statistical analysis

SPSS 11.0 software was used for the statistical analysis of the data. Correlation coefficients were estimated between and among values obtained in the ED and the laboratory by both the Pearson and Spearman methods (given the asymmetries in the variables). The linear fit was assessed by the *R*^2^ value. *P* values were estimated using a one-tailed test. Diabetes diagnoses were determined in the laboratory and ED, and the sample was stratified by age (>/<40 years). The diagnostic efficacy of the HbA1c value and glucose level in the blood was assessed for different cut-offs using ROC curves, and the sensitivity, specificity, and positive and negative predictive values were estimated. The degree of agreement was established with the kappa coefficient. The relationship was assessed with a chi-square test.

## Results

### Patient characteristics

A total of 187 patients that fulfilled inclusion criteria agreed to participate and, with all data available, were examined. Of them, 101 were men, with a median age of 64, interquartile range (IQR) of 66 years, and mean age of 57.1 ± 19.2 years (95 % CI 54.3–59.9 years) with a range of 18–84 years. Their demographic and clinical data are described in Table 2.

**Table 2:** Demographic, clinical, and analytical data

	*n* = 187
Age, years (mean ± SD)	57.1 ± 19.2
Male, *n* (%)	101 (54)
Emergency room diagnosis, *n* (%)	
Neurological	
Stroke	6 (3.2)
Epileptic seizure	2 (1.1)
Headache	3 (1.6)
Other	2 (1.1)
Cardiorespiratory	
COPD	5 (2.7)
Respiratory infection	16 (8.6)
Asthma	2 (1.1)
Chest pain	10 (5.3)
Cardiac arrhythmia	14 (7.5)
IHD	6 (3.2)
CHF	12 (6.4)
Other	9 (4.8)
Gastrointestinal	
Biliary diseases	6 (3.2)
GB	2 (1.1)
Abdominal pain	17 (9.1)
AGE	7 (3.7)
Appendicitis	3 (1.6)
Other	9 (4.8)
Renal	
UTI	5 (2.7)
Other	4 (2.2)
Psychiatry	
Anxiety	2 (1.1)
Other	5 (2.7)
Gynaecology	3 (1.6)
Musculoskeletal	15 (8)
Other	28 (15)
Diabetes prevalence, *n* (%)	
Prior known DM	32 (17.1)
Prior undiagnosed DM	10 (5.4)
Unknown DM	11 (5.9)
Total estimated DM	51 (28.5)
Capillary blood glucose in ED (mean + SD), mg/dL	114.8 ± 44.9
Laboratory blood glucose (mean + SD), mg/dL	117.3 ± 42.1
Capillary HbA1c in ED (mean + SD), % units	5.78 ± 1.26
Laboratory HbA1c (mean ± SD), % units	6.10 ± 1.12

*IHD* ischaemic heart disease, *COPD* chronic obstructive pulmonary disease, *AGE* acute gastroenteritis, *GB* gastrointestinal bleeding, *CHF* chronic heart failure, *UTI* urinary tract infection, *SD* standard deviation, *HbA1c* glycated haemoglobin

### Glucose and HbA1c

**Fig. 1:**
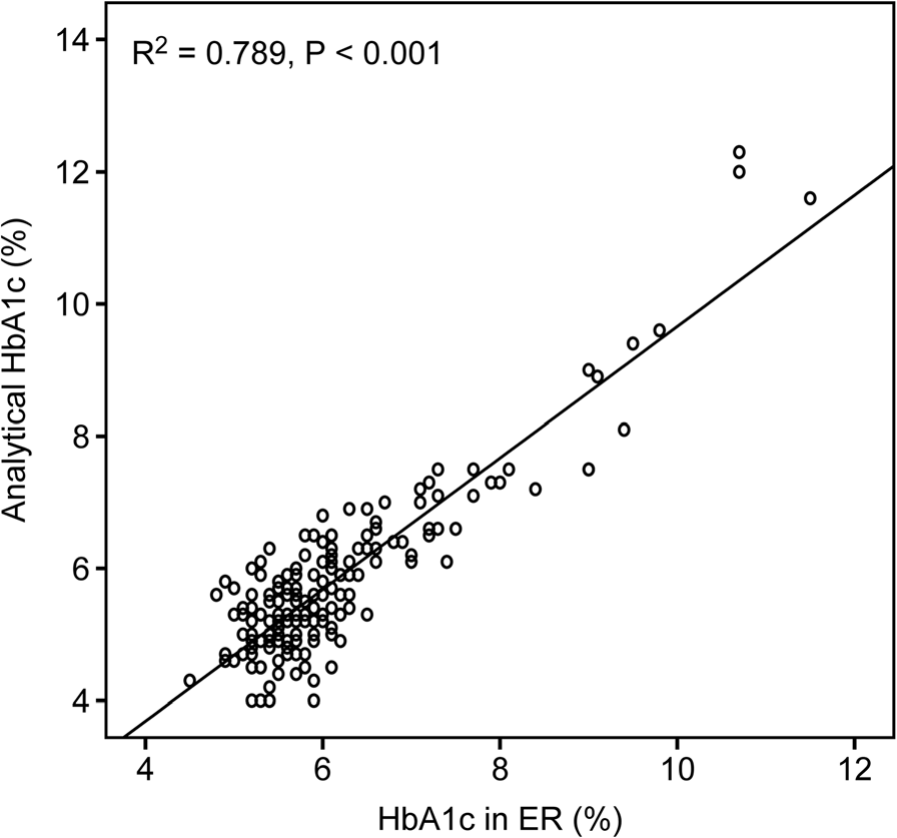
Capillary and laboratory haemoglobin A1c (*HbA1c*) correlation

The capillary blood glucose level (mean ± SD) was 114.8 ± 44.9 (95 % CI 108.3–121.3) mg/dL. The laboratory blood glucose level (mean ± SD) was 117.3 ± 42.1 (95 % CI 110.9–123.7) mg/dL. The capillary HbA1c value was 5.78 ± 1.26 % (95 % CI 5.60–5.97 %), with a range of 4.00–12.30 %, and the laboratory level was 6.10 ± 1.12 % (95 % CI 5.94–6.26 %), with a range of 4.50–11.50 %. The correlations between the measurements in the ED and laboratory were very high in both cases (glucose: *R*^2^ = 0.739, *P* < 0.0001; HbA1c: *R*^2^ = 0.789, *P* < 0.001) and are described in Fig. 1.

### Diagnosis of diabetes

Thirty-two subjects (17.1 %) stated a *prior known diagnosis of DM*. Using the 2015 ADA criteria for the diagnosis of DM (plasma glucose > 126 mg/dL and/or HbA1c > 6.5 %) and previous clinical history data, ten more cases were revealed (*prior undiagnosed DM*) (5.4 %). The total number of patients who met any criteria for *prior DM* was 42, for a 22.5 % prevalence rate (95 % CI 16.4–28.5 %). The capillary HbA1c values were useful in detecting 11 additional cases of *unknown DM* (5.9 %).

### Degree of control of prior DM

In patients with prior DM (*n* = 42), the HbA1c values were >7 % (61.9 %), >8 % (28.6 %), and >9 % (16.7 %) by the hospital laboratory and >7 % (47.6 %), >8 % (19.0 %), and >9 % (11.9 %) by capillary testing.

### Diagnostic efficacy of HbA1c and glucose levels

**Fig. 2:**
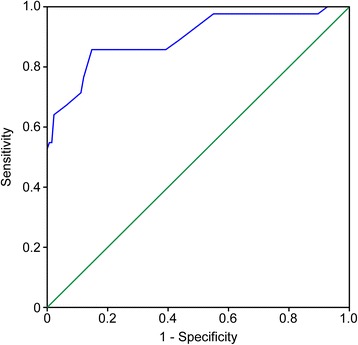
Diagnostic efficacy (sensitivity and specificity) for different cut-off values of capillary HbA1c assessed by receiver operating characteristic (ROC) curve (see also Table 2)

The ROC curve of the capillary HbA1c value measured in the ED had an area under the curve (AUC) of 0.897 (95 % CI 0.835–0.960, *P* < 0.0001) (Fig. 2). Using 6 % as the cut-off and cross-referencing with the DM diagnosis, we obtained a sensitivity (*S*) of 85.7 %, positive predictive value (PPV) of 63.2 %, specificity of 85.3 % (122 of 143), and negative predictive value (NPV) of 95.3 % (122 of 128) (*χ*^2^ = 76.83; 1 gl; *P* < 0.000; kappa = 0.631). According to coordinates of the curve, the 6 % cut-off seemed to best balance the sensitivity and specificity, with both greater than 85 % (Table 3). A previous study in acutely ill patients with random hyperglycaemia at hospital admission showed that a 6.0 % laboratory HbA1c level was 100 % specific (14/14) and 57 % sensitive for diabetes diagnosis [11].

**Table 3:** Diagnostic efficacy (sensitivity and specificity) for different cut-off values of capillary HbA1c assessed by receiver operating characteristic (ROC) curve

HbA1c (%)	Sensitivity	1: specificity
3.000	1.000	1.000
…	…	…
5.550	0.857	0.392
5.650	0.857	0.308
5.750	0.857	0.273
5.850	0.857	0.245
5.950	0.857	0.182
*6.050*	*0.857*	*0.147*
6.150	0.762	0.119
6.250	0.714	0.112
6.350	0.690	0.084
6.450	0.667	0.056
6.550	0.643	0.021
6.650	0.571	0.014
6.750	0.548	0.014
6.850	0.548	0.007
6.950	0.524	0.000
7.050	0.476	0.000
7.150	0.429	0.000

Diagnostic efficacy (sensitivity and specificity) for different cut-offs value of capillary HbA1c assessed by ROC (Receiver Operating Characteristic) curve. The best diagnostic performance is achieved with a cutoff of 6 % (marked in italics)

## Discussion

An early diagnosis of DM remains an unsolved challenge. Scientific data repeatedly confirm the need for an early treatment of DM to avoid the development of complications. However, the Di@bet.es study has shown that the prevalence of DM in the adult Spanish population is 13.8 %, with a distribution of 7.8 % of known DM and 6 % of unknown DM [12]. This rate of undiagnosed DM is clearly higher than that described in other countries [13] and highlights the importance of screening and early detection [14]. Current guidelines recommend diabetes screening in adults over 45 years of age and/or those who have a body mass index greater than 25 kg/m^2^ and with any additional known risk factor for DM [6]. The recommended tests for screening are the same as those used for diagnosis and include fasting plasma glucose (FPG), HbA1c level, and/or an oral glucose tolerance test (OGTT). The latter test is inconvenient, expensive, and complex, for which it is seldom used in clinical practice. Determination of blood glucose is limited by the influence of multiple factors (prior intake, drugs, stress, etc.). HbA1c measurement, although more expensive than blood glucose determination, provides information on the glycaemic environment during the previous 2 to 3 months.

Several epidemiologic studies and meta-analyses have reported that diabetes screening in the population over 40 years of age and in high-risk individuals (those with a family history of diabetes and/or hypertension) is cost-effective [15]. Determination of HbA1c is considered the best test, with an intermediate cost between measuring the FPG and the OGTT [15]. However, screening access by HbA1c testing is limited, especially in socioeconomically deprived areas, unless it is performed at primary care consultations [16]. Taking advantage of situations in which people come to the hospital can be a “window of opportunity” for the detection of DM. The population of adults treated in the ED, as described in our sample, would mostly belong to this high-risk group of undiagnosed DM patients [17]. In our sample, 22.5 % of adult patients who were treated at the ED met the DM criteria and another 5.9 % met the criteria for newly diagnosed DM. This finding is consistent with previous studies [7, 18, 19]. HbA1c levels are also an indicator of future DM and may be useful for defining personalized prevention strategies [20].

Hyperglycaemia is a risk marker for hospital mortality [1]. However, stressors and lack of fasting may influence the interpretation of a single measurement of blood glucose in the laboratory and/or ED. In addition, optimal thresholds for DM screening in ED have not been determined. Capillary HbA1c levels in the ED can be used to detect patients with poor glycaemic control who are at risk of acute and chronic complications [21–23]. Among the patients with DM who were included in our study, 47.6 % had an HbA1c value above 7 % (the accepted target of good control) and 19.0 % had clearly insufficient DM control (over 8 %). In the entire sample, 10.8 % of the patients showed levels above 7 % and 4.3 % had levels higher than 8 %.

Our study indicates that measurement of HbA1c by capillary point-of-care testing could provide considerable advantages in the detection of DM, including immediacy of the result, and the possibility of using the information to guide medical treatment. Clinical practice guidelines recognize that there are no randomized trials demonstrating improved outcomes using HbA1c levels to assist in the diagnosis of diabetes or to guide the glycaemic management of inpatients with known diabetes. However, they unanimously agreed on the practical utility of this strategy based in the consensus opinion [24]. The issue of a possible cost reduction can be addressed by future studies. These advantages are especially applicable to care in an ED. HbA1c information could modify the treatment indicated for the symptoms that brought the patient to the ED. Additionally, in patients with both known DM and newly diagnosed DM, capillary HbA1c information can be useful in determining the most appropriate antidiabetic treatment (i.e., use of oral agents vs. insulin).

Major limitations of our study are the inclusion of a single medical institution and the possibility of a selection bias. To limit this constraint, we recruited all adult patients with no limitations other than their consent or use of hyperglycaemic medications such as corticosteroids. In addition, haemoglobinopathies are a known cause of erroneous results in determining HbA1c levels; however, the prevalence of these diseases in our population was very low.

## Conclusions

Determination of capillary HbA1c levels in the ED is a reliable, fast, and simple system for the detection of unknown and poorly controlled DM. Due to the need to improve the early detection of DM and the appropriate selection of antidiabetic therapy, our results suggest that capillary HbA1c represents an important diagnostic tool in the ED. Future studies should investigate if the use of capillary HbA1c measurement in the ED can improve clinical outcome, reduce complications, and guide treatment selection in patients with hyperglycaemia and diabetes.

## Abbreviations

DM: diabetes mellitus
ED: emergency department
FPG: fasting plasma glucose
HbA1c: haemoglobin A1c
OGTT: oral glucose tolerance test
SD: standard deviation
